# COVID-19 Vaccine Hesitancy among Arab Americans

**DOI:** 10.3390/vaccines10040610

**Published:** 2022-04-14

**Authors:** Mira H. Kheil, Deepti Jain, Jamil Jomaa, Brandon Askar, Yasmeen Alcodray, Shatha Wahbi, Salar Brikho, Ali Kadouh, Deanna Harajli, Zain N. Jawad, Ziad Fehmi, Malaak Elhage, Tala Tawil, Omar Fehmi, Suma J. Alzouhayli, Deema Ujayli, Noor Suleiman, Omar Kazziha, Rawan Saleh, Evi Abada, Anita Shallal, Seongho Kim, Vijaya Arun Kumar, Marcus Zervos, Michele L. Cote, Rouba Ali-Fehmi

**Affiliations:** 1 Department of Pathology, Wayne State University School of Medicine and Detroit Medical Center, 3990 John R. Road, Detroit, MI 48201, USA; mkheil@med.wayne.edu (M.H.K.); deepti.jain@wayne.edu (D.J.); hk0146@wayne.edu (T.T.); srawan02@gmail.com (R.S.); gs5839@wayne.edu (E.A.); 2 School of Medicine, Wayne State University, 4201 Street Antoine, Detroit, MI 48201, USA; gs8226@wayne.edu (J.J.); fv7083@wayne.edu (B.A.); ga6296@wayne.edu (Y.A.); hf8920@wayne.edu (S.W.); gb1930@wayne.edu (S.B.); ga9451@wayne.edu (A.K.); hc3551@wayne.edu (D.H.); zain.jawad@med.wayne.edu (Z.N.J.); suma.alzouhayli@med.wayne.edu (S.J.A.); noor.suleiman@med.wayne.edu (N.S.); 3 College of Literature, Science and the Arts, The University of Michigan, 101 N Main Street, Ann Arbor, MI 48104, USA; zfehmi@umich.edu (Z.F.); ofehmi@umich.edu (O.F.); 4 College of Liberal Arts and Sciences, Wayne State University, 4841 Cass Avenue, 2155 Old Main, Detroit, MI 48201, USA; melhage@wayne.edu; 5 College of Human Medicine, Michigan State University, 965 Fee Road A110, East Lansing, MI 48824, USA; ujaylide@msu.edu; 6 College of Education, Wayne State University, 5425 Gullen Mall, Detroit, MI 48202, USA; gq0480@wayne.edu; 7 Division of Infectious Diseases, Henry Ford Hospital, 2799 W. Grand Blvd, CFP 303, Detroit, MI 48202, USA; ashalla2@hfhs.org (A.S.); mzervos1@hfhs.org (M.Z.); 8 Biostatistics Core, Karmanos Cancer Institute, Department of Oncology, Wayne State University School of Medicine, 4100 John R. Road, Detroit, MI 48201, USA; kimse@karmanos.org; 9 Population Sciences and Disparities Research, Karmanos Cancer Institute, Department of Oncology, Wayne State University School of Medicine, 4100 John R. Road, Detroit, MI 48201, USA; cotem@karmanos.org; 10 Department of Emergency Medicine, Wayne State University School of Medicine and Detroit Medical Center, 4201 Saint Antoine, Suite 6F UHC, Detroit, MI 48201, USA; vkumar@med.wayne.edu

**Keywords:** COVID-19 vaccine, Arab Americans, vaccine hesitancy, Arab American health

## Abstract

(1) Background: Coronavirus disease-2019 (COVID-19) vaccines have a significant impact on reducing morbidity and mortality from infection. However, vaccine hesitancy remains an obstacle in combating the pandemic. The Arab American (AA) population is understudied; thus, we aimed to explore COVID-19 attitudes within this community. (2) Methods: This was a cross-sectional study. An anonymous online survey was distributed to members of different AA associations and to the community through the snowball method. (3) Results: A total of 1746 participants completed the survey. A total of 92% of respondents reported having received at least one dose of a COVID-19 vaccine. A total of 73% reported willingness to receive a booster, and 72% plan to give their children the vaccine. On multivariate analysis, respondents were more likely to be vaccine-hesitant if they were hesitant about receiving any vaccine in general. They were less likely to be vaccine-hesitant if they were immigrants, over the age of 40, up to date on their general vaccination and if they believed that COVID-19 vaccines are safe and effective in preventing an infection. The belief that all vaccines are effective at preventing diseases was also associated with lower hesitancy. (4) Conclusions: This sample of AAs have higher vaccination rates and are more willing to vaccinate their children against COVID-19 when compared to the rest of the population. However, a reemergence of hesitancy might be arising towards the boosters.

## 1. Introduction

Severe acute respiratory coronavirus 2 (SARS-CoV-2) is a highly infectious virus that causes Coronavirus disease 2019 (COVID-19) [1]. COVID-19 abruptly spread internationally, exposing the world to a potentially deadly virus. Despite public health implementations, including masks mandates, lockdowns, and social distancing, millions of lives have been lost, and additional measures needed to be taken [2]. Vaccines were rapidly developed, and although vaccination does not offer full protection against COVID-19 infection [3], it has had a significant impact on controlling COVID-19 outbreaks and improving prognosis. In the United States (US), COVID-19 vaccines decreased the overall attack rate from 9% to 4.6% within 300 days [4]. Throughout the same period, adverse outcomes decreased by 63.5%, non-intensive care unit hospitalization by 65.6%, and deaths by 69.3%, making preventative vaccines the safest and most cost-effective way to combat this pandemic [2,4]. Unfortunately, these vaccines were met with resistance from a portion of the population. Vaccine hesitancy, defined as reluctance to receive vaccines despite their availability, is one of the top 10 global health threats, according to the World Health Organization (WHO) [5].

Racial and ethnic differences contribute to vaccine attitudes, and levels of vaccine hesitancy and acceptance exist across different communities [6]. COVID-19 vaccine acceptance rates in Arab countries are lower than rates in the general US population (32.3% compared to 58–69%, respectively) [7,8]. Vaccine hesitancy among the Arab population living in Arab countries ranges between 20 to 41% [9,10,11]. Hesitancy in this group was correlated with concerns about long-term side effects and a lack of thorough evaluation or trust in the Food and Drug Administration (FDA) approval process [10]. Residents of Arab countries are also reported to be more hesitant due to a lack of trust in national healthcare, lack of sufficient studies, or the perceived lack of need due to the high number of people who have already been infected in their country [7]. However, a study by Kaadan et al. reported a higher acceptability rate of the vaccine in Arabs who reside outside their home countries compared to those who reside in their home countries [12]. They argued that vaccine attitudes are influenced by the general population where the individual lives.

To explore this further, we aimed to conduct a study categorizing and identifying reasons for vaccine hesitancy among the adult Arab American population using an anonymous online survey.

## 2. Materials and Methods

### 2.1. Study Design

This was a cross-sectional study using an anonymous online survey created on SurveyMonkey (San Mateo, CA, USA). The survey was distributed electronically to the Arab Community Center for Economic and Social Services (ACCESS) employees and patients, the National Arab American Medical Association (NAAMA), and NAAMA NextGen members, and informal networks serving the Arab community. ACCESS is the largest Arab American community nonprofit in the US, offering a wide range of social, economic, health, and educational services to the population. NAAMA and NAAMA NextGen are all-inclusive associations that serve as the voice of all Arab American healthcare professionals and students while providing educational, philanthropic, and service activities. The survey was also shared among students at Wayne State University and University of Michigan and with patients in the Wayne County community clinic in Hamtramck. Electronic flyers with the survey link were shared on social media platforms of NAAMA, NAAMA NextGen, and ACCESS and through messaging applications. The survey link was further shared through the snowball method [13]. It was administered between 18 October 2021 and 29 November 2021) to adults aged 18 years or older who were able to provide informed consent.

We defined vaccine hesitancy as “the reluctance or refusal to vaccinate despite availability of vaccines” as per the WHO [5]. Arabs born in their country of origin who later moved to the US are described as immigrants. Arab countries in the Middle East and North Africa (MENA) region were divided into three culturally and geographically similar regions: Greater Maghreb (Morocco, Mauritania, Algeria, Tunisia, Libya), Fertile Crescent (Lebanon, Syria, Palestine, Egypt, Iraq, Jordan), and Arabian Peninsula (Saudi Arabia, United Arab Emirates, Qatar, Oman, Bahrain, Kuwait, Yemen). 

### 2.2. Ethical Considerations

Participants provided informed consent, and their privacy was maintained by the use of anonymous surveys. The Institutional Review Boards of Wayne State University School of Medicine described the study as a non-human subjects research study. The Research Review process at ACCESS provided a letter of support. Each Board of Directors of ACCESS, NAAMA, and NAAMA NextGen approved the collection of their employees’ and patients’ emails.

### 2.3. Survey Items

The demographic information in the survey includes age, gender, country of origin (Arab), immigration status, the highest level of education, and yearly gross household income. Our survey also collected information about prior vaccine behaviors, respondents’ belief in safety, effectiveness, and hesitancy towards receiving Center for Disease Control and Prevention (CDC) recommended vaccines and current vaccination status. Participants’ attitudes towards COVID-19 and COVID-19 vaccines were further explored by including items about having had a COVID-19 positive diagnosis, receiving at least one dose or both (if applicable) of the COVID-19 vaccine, personal belief in its effectiveness and safety, willingness to encourage family or friends to become vaccinated, plans to vaccinate their children when available, and willingness to receive the COVID-19 vaccine booster.

### 2.4. Statistical Methods

Categorical variables were reported by count and frequency, and median and range were used to summarize continuous variables. Vaccine hesitancy was reported as binary (Yes vs. No, No as reference) using the question “Have you received at least one dose of a COVID-19 vaccine?” where we defined vaccine hesitancy as a “No” response. Univariate and multivariate logistic regression analyses were performed to identify associations between vaccine hesitancy and respondents’ characteristics and their beliefs about vaccines and vaccination. We selected covariates for multivariable analysis a priori, based on potential confounders identified in similar research [7,14]. All 14 variables in Table 1 were included for both univariate and multivariate analyses, except for three variables: “I would encourage my family or friends to get COVID-19 vaccine,” “When available, I plan to have my kid(s) receive the COVID-19 vaccine,” and “Have you received at least one dose of a COVID-19 vaccine?” To further avoid underpowered multivariate analysis, we reduced the number of levels of each variable as small as possible while ensuring the events per variable at least to be five [15]. Odds ratios and 95% confidence intervals were estimated from the logistic regression models, with *p*-value ≤ 0.05 considered statistically significant. For categorical variables with three or more levels, we also calculated global *p*-values using likelihood ratio tests.

## 3. Results

### 3.1. Participant Characteristics and COVID-19 Beliefs

We received a total of 1746 survey responses. The majority (51%) of participants were between the ages of 18 and 29 (n = 883). Only 4% (n = 65) were above the age of 70 years. A total of 55% (n = 960) of the respondents were females and 45% (n = 779) were males. The vast majority of the participants were originally from the Fertile Crescent (n = 1667, 95%). Half were immigrants (n = 871, 50%) and 43% (n = 753) were born and raised in the US.

29% of the participants had received a Bachelor’s degree (n = 505) and a quarter of them hold Doctorate degrees (n = 442, 25%) as the highest level of education. One-third of respondents (n = 572, 33%) have a yearly household income of over $150,000.

Most participants (n = 1587, 91%) reported believing that vaccination, in general, is effective at preventing diseases, and 72% (n = 1254) believed that all CDC vaccine recommendations are safe. The majority (75%, n = 1309) denied being hesitant about receiving any vaccine, and 90% (n = 1570) reported being up-to-date on their vaccinations. Nearly a quarter of the participants were diagnosed with COVID-19 (23%, n = 407) at some point. The majority (80%, n = 1402) believed that the COVID-19 vaccines are effective at preventing infection, and similarly, 78% (n = 1364) believed it was a safe vaccine. Most respondents (n = 1480, 85%) would encourage their family and friends to receive the COVID-19 vaccine, and 72% (n = 752) plan to give it to their children when available among those who have children (n = 1049).

Around half (n = 892, 51%) of survey respondents reported being afraid of the new COVID-19 variants, and the majority of them had already received at least one dose of the vaccine (n = 1603, 92%) (Table 1).

### 3.2. COVID-19 Vaccine Compliance

Among the participants who had received at least one dose of the COVID-19 vaccine, 61% (n = 982) received the Pfizer-BioNTech vaccine, 33% (n = 530) received the Moderna vaccine, and 4% (n = 62) received the Johnson & Johnson vaccine. Nearly all (95%) either already received or planned on receiving their second dose, if applicable. A lower percentage of participants were planning on receiving the booster (n = 1172, 73%) (Table 2).

### 3.3. COVID-19 Vaccine Hesitancy

Among the 105 respondents who did not receive a COVID-19 vaccine, 86% (n = 90) reported it was due to safety concerns and side effects of the vaccine. Most (70%, n = 73) reported distrust in the healthcare system, vaccines, and the government. An additional 21% (n = 22) were hesitant due to religious or personal beliefs, and 7% (n = 7) did not get it because they had already been infected with COVID-19 (Table 3).

### 3.4. Factors Associated with Vaccine Hesitancy

Results showed that respondents were more likely to be vaccine-hesitant if they were hesitant towards receiving any vaccination in general, and this result was significant in both univariate (odds ratio (OR), 9.061; 95% CI, 5.902 to 13.863; *p* < 0.001) and multivariate (OR, 2.893; 95% CI, 1.429 to 5.870; *p* = 0.003) analyses. In addition, participants with a positive COVID-19 diagnosis in the past were more likely to be hesitant about the vaccine compared to those who had not contracted the disease. However, while this observation was statistically significant on univariate analysis (OR, 3.428; 95% CI, 2.293 to 5.121; *p* = 0.001), it was not significant on multivariate analysis (OR, 1.882; 95% CI, 0.983 to 3.601; *p* = 0.055).

When compared to men, we found that women were more likely to be vaccine-hesitant on univariate analysis (OR, 1.714; 95% CI, 1.135 to 2.639; *p* < 0.012); however, this observation did not hold statistical significance in multivariate analysis (OR, 1.741; 95% CI, 0.880 to 3.545; *p* = 0.117).

Furthermore, respondents who were immigrants were less likely to be vaccine hesitant compared to those who were born and raised in US, and this finding was statistically significant for both univariate (OR, 0.362; 95% CI, 0.231 to 0.555; *p* < 0.001) and multivariate (OR, 0.380; 95% CI, 0.182 to 0.767; *p* = 0.008) analyses. Vaccine hesitancy was found to be significantly less in participants aged 50+ years on univariate analysis (OR, 0.223; 95% CI, 0.098 to 0.441; *p* < 0.001), and of ages 40–49 years on multivariate analysis (OR, 0.181; 95% CI, 0.043 to 0.639; *p* < 0.013), compared to those age 18–29 years. In addition, vaccine hesitancy was found to be significantly less on univariate analysis in people who had a graduate degree (OR, 0.353; 95% CI, 0.189 to 0.682; *p* < 0.001), those with household income greater than $100,000 yearly (OR, 0.389; 95% CI, 0.221 to 0.695; *p* < 0.001), those that believed that all CDC recommended vaccines are safe (OR, 0.049; 95% CI, 0.027 to 0.083; *p* < 0.001) and among those who were afraid of the new COVID-19 variants (OR, 0.257; 95% CI, 0.159 to 0.402; *p* < 0.001). However, these associations were not statistically significant on multivariate analysis.

Participants were also less likely to be vaccine hesitant if they were up to date with their vaccines in general (OR, 0.108; 95% CI, 0.070 to 0.169; *p* < 0.001 and OR, 0.359; 95% CI, 0.170 to 0.756; *p* < 0.007 on univariate and multivariate analysis respectively), if they believed that vaccines prevent diseases (OR, 0.058; 95% CI, 0.037 to 0.091; *p* < 0.001 and OR, 0.473; 95% CI, 0.224 to 0.996; *p* < 0.049 on univariate and multivariate analysis respectively), if they believed that the COVID-19 vaccine is effective at preventing COVID-19 (OR, 0.022; 95% CI, 0.011 to 0.038; *p* < 0.001 and OR, 0.136; 95% CI, 0.055 to 0.310; *p* < 0.001 on univariate and multivariate analysis respectively) and if they believed that the COVID-19 vaccine is safe (OR, 0.019; 95% CI, 0.009 to 0.036; *p* < 0.001 and OR, 0.144; 95% CI, 0.052 to 0.366; *p* < 0.001 on univariate and multivariate analysis respectively). These findings held true on both univariate and multivariate analyses (Table 4).

## 4. Discussion

Our study reports factors associated with COVID-19 vaccine hesitancy among AAs living in the US. Hesitancy was lower among older individuals, those born outside of the US, and among those that believe overall that vaccines are safe, including the COVID-19 vaccine. These findings can be compared to prior studies before the COVID-19 vaccine was widely available to the general public [12,14,16,17].

A previous survey on COVID-19 vaccine hesitancy among AAs was conducted between May and September of 2020 by Abouhala et al. [14]. It is important to note that at the time, vaccines were still not available, and respondents were reporting their future intentions on vaccination. It had also been less than a year into the pandemic, and its progression was still unclear. Currently, we are entering the third year of the pandemic, and vaccines are widely available. A COVID-19 vaccine is no longer theoretical, and the public is more familiar with the virus itself and how it behaves. We expected these differences to change the population’s willingness to receive a vaccine, and hence we anticipated a change in reported outcomes. 

The national pooled prevalence rate of COVID-19 vaccine hesitancy for adult Americans was reported to be 26.3% [18]. However, vaccine attitudes are fluid, and hesitancy is likely to wane over time [19]. A massive online survey in the US showed that COVID-19 vaccine hesitancy decreased from 25.4% in January 2021 to 16.6% in May 2021 as the pandemic progressed [17]. Similarly, lower vaccine hesitancy trends among AAs can be appreciated. In the study by Abouhala et al., only 56.7% of AAs reported an intention of receiving the vaccine, and 35.7% were unsure [14]. Our results revealed that 92% of our respondents have already received one shot of the vaccine, suggesting increased confidence among a community that was previously hesitant.

Our results also showed higher vaccine hesitancy among females on univariate analysis. Even though this finding was not significant in multivariate analysis, it is still worthy of noting as it is consistently reported in the literature and is prominent even among healthcare workers [14,20]. Female vaccine hesitancy has an implication for public health as it stems from the misconception that the vaccine may affect fertility [21]. It has also been previously reported that AA women have lower rates of vaccine compliance in general, especially if they were foreign-born [22].

We found adults over the age of 40 to be more vaccine confident than the younger population, which is consistent with what is reported among the American population, where adults over the age of 50 were less likely to be vaccine-hesitant [23]. This is contrasted with older studies on the AA population, where older individuals were more hesitant [14]. We attribute this finding to the increased severity and higher mortality of COVID-19 in older individuals, potentially increasing fear among this population and leading to more vaccine compliance.

Immigration status was also significantly associated with vaccine attitudes among AAs, such that those who had immigrated were more vaccine confident than American-born Arabs. Similar results can be noted when compared to other immigrants. In a study on COVID-19 vaccine attitudes among immigrants in California, adults of immigrant families were more likely to receive the vaccine than non-immigrants, reporting higher levels of trust in public health officials and healthcare providers [16]. It is important to note here that it is the immigration status that is related to hesitancy rather than the ethnic group. In a study on non-immigrant Hispanics and African Americans, COVID-19 vaccine hesitancy was higher among these groups (30.2% and 41.6%, respectively) than the national average (26.3%) [18]. One of the major predictors of these communities’ hesitancy was medical mistrust and a history of racial discrimination [18].

Our most significant results on multivariate analysis demonstrated higher COVID-19 vaccine hesitancy in individuals who reported being hesitant towards receiving any vaccine and higher COVID-19 vaccine confidence in those who were up to date with their general vaccines and those who believed that vaccines are effective at disease prevention. AAs with positive attitudes towards the safety and efficacy of the COVID-19 vaccine were less likely to be hesitant about the vaccine. These findings were consistent with several other studies [12,17,23]. In a systematic review of factors influencing vaccine hesitancy in the US, the likelihood of pursuing vaccination was significantly associated with the efficacy and perceived harmful effects of the COVID-19 [23].

Safety concerns and side effects of the vaccine were the most commonly reported reason for COVID-19 vaccine hesitancy in our study (90%) and in the literature, followed by distrust in the healthcare system, vaccines, and the government [17]. Religious and personal beliefs were other reasons that contributed to our sample’s vaccine hesitancy. In the study by Abouhala et al., an association between religiosity and vaccine hesitancy was established within the AA population [14]. However, this association is also prominent in the American population, where conservatism significantly predicted vaccine refusal [23]. Finally, the belief that previous COVID-19 infection is a reason not to receive the vaccine is still common, as evident from our survey. With the spread of new variants and the need for boosters, the public should be properly educated on the scientific evidence behind the necessity for further vaccination and vaccine safety, especially with non-factual information and anti-vaccine campaigns being prominent on social media [24].

Similarly, the receipt of previous doses of the vaccine might give a false sense of protection, contributing to a decrease in receiving boosters. When asked about the intention to receive a booster, only 73% of our previously vaccinated respondents reported planning to take it. The reemergence of hesitancy towards the booster appears to be present within the American population, too, such that 38.2% reported being booster dose hesitant [25]. The public’s confidence in the COVID-19 vaccine efficiency seems to be decreasing as they learn that more boosters are needed. Pandemic fatigue could also be contributing to less compliance with receiving additional vaccine doses. These statistics highlight the need for continuous education and local campaigning on the importance of boosters.

Our participants were also found to be less likely to be vaccine-hesitant if they reported being afraid of the new COVID-19 variants. This was expected as health-related fears regarding COVID-19 were shown to promote higher vaccine acceptance [26]. Mangla et al. investigated the attitudes and beliefs towards the COVID-19 vaccine and the new variants in six countries (USA, Bangladesh, Columbia, India, Malaysia, and Zimbabwe) [27]. A total of 49.68% of the respondents had not taken the vaccine yet, although 92% claimed that they are aware of the new variants’ seriousness [27].

A controversial population during this pandemic is children. Although most children have lower risks of short-term complications with COVID-19 infection, deaths and hospitalizations have been reported, and protection of this group remains a public health concern [28]. COVID-19 vaccines were approved by the FDA in October 2021 for children aged 5 years and older [29]. In our sample, 73% of participants plan on giving their children a COVID-19 vaccine. Comparing our results to the American population reveals higher hesitation among Americans towards vaccinating children. Among vaccinated American parents, 20.1% do not plan on vaccinating their children, and 14.5% were unsure [25]. Among unvaccinated parents, those numbers are higher such that 61.4% reported not intending to vaccinate their children and 20.4% were unsure [25]. In another study, Ruggiero et al. reported that about half of US parents (49.45%) are willing to give the COVID-19 vaccine to their children. However, although they have a positive attitude towards the vaccine, 69.53% disclose more hesitancy toward the COVID-19 vaccine than other previous child vaccinations. These results were comparable to a study by Szilagyi et al., assessing the likelihood of 1745 US parents vaccinating their children. For 46% of children, parents were “very likely” or “likely” to have their child receive the vaccine. For 33% of children, parents were “very unlikely” to vaccinate them, 9% were “somewhat unlikely”, and 12% were “unsure” [30]. Notably, vaccine hesitancy among parents decreased if their children were older [31]. Their overall vaccine hesitancy for themselves is highly predictive of their hesitancy towards vaccinating their children [30,31]. Hesitancy towards vaccinating their children was mostly due to concerns about side effects (61.5%) and about vaccine safety (48.96%) [30]. Parents’ factors associated with a higher likelihood to vaccinate their children include parents of older children, parents who were Asian American or Hispanic, had a bachelor’s degree or higher education, had already received or are likely to receive the COVID-19 vaccine. Interestingly, parents consider their children’s doctor the most trusted source, rather than the CDC or government [30].

It is worth highlighting some of our study’s unique strengths. To our knowledge, this is the largest study to investigate the AA population’s attitudes towards the COVID-19 vaccine. It is also the first to inquire about attitudes regarding the new variants, receiving boosters, and vaccinating children. The pandemic has been very dynamic, and our study provides an updated, current snapshot of the AA population’s attitudes. Furthermore, we received a near-complete response to our survey questions due to our easily understood survey design. The electronic nature of our survey also made it easier to share around the states and reach a higher number of respondents. Our results give insight into the AA population and may be significant to create public health interventions to increase vaccine acceptance.

Our study did have some limitations. As this was a voluntary study, those who chose to participate may not be representative of the general population. It is also possible that this recruitment strategy introduced selection bias, with our respondents systematically differing from the population of interest. Thus, our 92% vaccination rate may overestimate the true percentage of the AA population. More than half of our participants were of younger age (18–29 years old). This could be reflective of the AA population, which is increasingly becoming younger but could have also been altered by the use of electronic surveys, which might not have reached older individuals. About one-third of our respondents had an annual income above $100,000, which is significantly higher than the general US population’s mean income of around $67,000 [32]. Median income and level of education are known predictors for vaccine confidence and may have skewed our study results [33,34]. Though several demographic and belief factors were taken into consideration in the multivariant analysis (Table 1), the respondents’ medical condition and prevailing health were not, thus creating a potential for a confounding bias. Respondents with several comorbidities would be less likely to be vaccine-hesitant and thus be a positive confounder. Another possible confounding factor could be that vaccine-confident subjects are more likely to agree to participate in the study than hesitant individuals. Finally, it is important to note that vaccine hesitancy is fluid and can easily vary with time and new research findings, so our results are a reflection of the current attitudes and are subject to change in the future.

## 5. Conclusions

At this point in the COVID-19 pandemic, AAs seem to have become less hesitant towards the COVID-19 vaccine and have a higher vaccination rate, and are more willing to vaccinate their children when compared to the rest of the population. However, a reemergence of hesitancy might be arising towards boosters. Thus, it is of utmost importance to continue to educate the community and develop targeted local campaigns that address their concerns and clarify their misconceptions.

## Figures and Tables

**Table 1:** Participant characteristics and beliefs and attitudes toward vaccinations.

Survey Question	All (N = 1746)
**What is your age?—no. (%)**	
18–29 years old	883 (51)
30–39 years old	250 (14)
40–49 years old	196 (11)
50–59 years old	223 (13)
60–69 years old	129 (7)
70+ years old	65 (4)
**What is your gender?—no. (%)**	
Male	779 (45)
Female	960 (55)
Other	3 (0)
Unknown	4 (0)
**What is your highest level of education?—no. (%)**	
Less than a high school degree	12 (1)
High school degree or GED	144 (8)
College	295 (17)
Associate’s degree	78 (4)
Bachelor’s degree	505 (29)
Master’s degree	270 (15)
Doctorate	442 (25)
**What is your country of origin?—no. (%)**	
Arabian Peninsula	64 (4)
Fertile Crescent	1667 (95)
Greater Maghreb	15 (1)
**Which of the following best describes you?—no. (%)**	
I was born and raised in the US	752 (43)
I am an immigrant	871 (50)
I am a refugee	33 (2)
Other	90 (5)
**What is your yearly household income?—no. (%)**	
<20 K	74 (4)
20 K–50 K	171 (10)
50 K–75 K	207 (12)
75 K–100 K	204 (12)
100 K–150 K	241 (14)
>150 K	572 (33)
Unknown	277 (16)
**Were you diagnosed with COVID-19?—no. (%)**	
No	1338 (77)
Yes	407 (23)
Unknown	1 (0)
**I believe that vaccinations in general are effective at preventing diseases: —no. (%)**	
Disagree	16 (1)
Neither agree nor disagree	110 (6)
Agree	1587 (91)
Unknown	33 (2)
**I believe that all CDC recommended vaccinations are safe: —no. (%)**	
Disagree	104 (6)
Neither agree nor disagree	355 (20)
Agree	1254 (72)
Unknown	33 (2)
**I am hesitant towards receiving any vaccinations (i.e., flu shot, tDAP, HPV, etc.): —no. (%)**	
Disagree	1309 (75)
Neither agree nor disagree	229 (13)
Agree	175 (10)
Unknown	33 (2)
**I believe I am up-to-date on my vaccinations in general: —no. (%)**	
Disagree	54 (3)
Neither agree nor disagree	89 (5)
Agree	1570 (90)
Unknown	33 (2)
**I believe that the COVID-19 vaccines are effective at preventing COVID-19: —no. (%)**	
Disagree	106 (6)
Neither agree nor disagree	202 (12)
Agree	1402 (80)
Unknown	36 (2)
**I believe that the COVID-19 vaccine is safe: —no. (%)**	
Disagree	69 (4)
Neither agree nor disagree	280 (16)
Agree	1364 (78)
Unknown	33 (2)
**I would encourage my family or friends to get COVID-19 vaccine: —no. (%)**	
Disagree	63 (4)
Neither agree nor disagree	165 (9)
Agree	1480 (85)
Unknown	38 (2)
**When available, I plan to have my kid(s) receive the COVID-19 vaccine: —no. (%)**	
Disagree	118 (7)
Neither agree nor disagree	123 (7)
Agree	752 (43)
Do not have any children	697 (40)
Unknown	56 (3)
**I am afraid of the new COVID-19 variants: —no. (%)**	
Disagree	323 (18)
Neither agree nor disagree	471 (27)
Agree	892 (51)
Unknown	60 (3)
**Have you received at least one dose of a COVID-19 vaccine? —no. (%)**	
Yes	1603 (92)
No	105 (6)
Unknown	38 (2)

**Table 2:** COVID-19 vaccination among those who had at least one dose of a COVID-19 vaccine.

Survey Question	N = 1603
**If yes, which shot did you receive?** **—** **no. (%)**	
Pfizer	982 (61)
Moderna	530 (33)
Johnson & Johnson	62 (4)
Other	10 (1)
Unknown	19 (1)
**If you took a COVID-19 vaccination that requires two shots (e.g., Moderna or Pfizer), have you received your second dose?** **—** **no. (%)**	
No, but plan on receiving 2nd dose	16 (1)
No, and I don’t plan on receiving 2nd dose	2 (0)
Yes	1507 (94)
Unknown	78 (5)
**When available, I plan on receiving a booster shot for COVID-19 vaccine: —no. (%)**	
Disagree	133 (8)
Neither agree nor disagree	239 (15)
Agree	1172 (73)
Unknown	59 (4)

**Table 3:** Reasons for COVID-19 vaccination hesitancy among respondents who had not received a COVID-19 vaccine.

Reasons—No. (%)	N = 143
Safety concerns/side effects	90 (86)
Distrust in healthcare system/Vaccines/government	73 (70)
Religious beliefs/Personal beliefs	22 (21)
Already had COVID-19	7 (7)

**Table 4:** Univariate and multivariate logistic analyses of risk factors associated with vaccine hesitancy (Yes vs. No, No as reference).

		Univariate ^a^			Multivariate ^b^	
	E/N	OR (95% CI)	*p*	E/N	OR (95% CI)	*p*
**What is your age?**			***0.001 ^c^***			***0.034 ^c^***
18–29 years old	70/857	Reference		55/701	Reference	
30–39 years old	17/246	0.835 (0.467, 1.413)	0.519	15/225	0.754 (0.305, 1.798)	0.531
40–49 years old	10/194	0.611 (0.291, 1.155)	0.157	5/169	0.181 (0.043, 0.639)	***0.013***
50+ years old	8/411	0.223 (0.098, 0.441)	***<0.001***	8/320	0.292 (0.090, 0.875)	***0.033***
**What is your gender?**						
Male	34/754	Reference		27/624	Reference	
Female	71/948	1.714 (1.135, 2.639)	***0.012***	56/791	1.741 (0.880, 3.545)	0.117
**What is your highest level of education?**			***0.004 ^c^***			0.076 *^c^*
High school degree or less	16/147	Reference		11/110	Reference	
College	60/859	0.615 (0.352, 1.133)	0.101	50/708	0.352 (0.117, 1.089)	0.064
Graudate degree	29/702	0.353 (0.189, 0.682)	***0.001***	22/597	0.730 (0.238, 2.362)	0.588
**What is your country of origin?**						
Arabian Peninsula/Greater Maghreb	8/76	Reference		6/57	Reference	
Fertile Crescent	97/1632	0.537 (0.265, 1.240)	0.109	77/1358	1.146 (0.284, 5.090)	0.853
**Which of the following best describes you?**			***<0.001 ^c^***			***0.002 ^c^***
I was born and raised in the US	69/733	Reference		54/615	Reference	
I am an immigrant	31/855	0.362 (0.231, 0.555)	***<0.001***	27/710	0.380 (0.182, 0.767)	***0.008***
Other	5/120	0.418 (0.144, 0.963)	0.066	2/90	0.082 (0.010, 0.401)	***0.006***
**What is your yearly household income?**			***0.001 ^c^***			0.785 *^c^*
<50 K	22/242	Reference		22/234	Reference	
50 K–100 K	31/397	0.847 (0.481, 1.516)	0.569	31/387	0.970 (0.413, 2.294)	0.944
>100 K	30/801	0.389 (0.221, 0.695)	***0.001***	30/794	0.768 (0.315, 1.891)	0.562
**Were you diagnosed with COVID-19?**						
No	54/1310	Reference		42/1099	Reference	
Yes	51/397	3.428 (2.293, 5.121)	***<0.001***	41/316	1.882 (0.983, 3.601)	0.055
**I believe I am up-to-date on my vaccinations in general:**						
Disagree or neutral	40/140	Reference		32/116	Reference	
Agree	65/1568	0.108 (0.070, 0.169)	***<0.001***	51/1299	0.359 (0.170, 0.756)	***0.007***
**I believe that vaccinations in general are effective at preventing diseases:**						
Disagree or neutral	48/123	Reference		38/94	Reference	
Agree	57/1585	0.058 (0.037, 0.091)	***<0.001***	45/1321	0.473 (0.224, 0.996)	***0.049***
**I believe that all CDC recommended vaccinations are safe:**						
Disagree or neutral	90/455	Reference		72/358	Reference	
Agree	15/1253	0.049 (0.027, 0.083)	***<0.001***	11/1057	0.497 (0.203, 1.174)	0.116
**I am hesitant towards receiving any vaccinations (i.e., flu shot, tDAP, HPV, etc.):**						
Disagree or neutral	59/1535	Reference		46/1277	Reference	
Agree	46/173	9.061 (5.902, 13.863)	***<0.001***	37/138	2.893 (1.429, 5.870)	***0.003***
**I believe that the COVID-19 vaccines are effective at preventing COVID-19:**						
Disagree or neutral	92/304	Reference		73/234	Reference	
Agree	13/1401	0.022 (0.011, 0.038)	***<0.001***	10/1181	0.136 (0.055, 0.310)	***<0.001***
**I believe that the COVID-19 vaccine is safe:**						
Disagree or neutral	95/345	Reference		75/268	Reference	
Agree	10/1363	0.019 (0.009, 0.036)	***<0.001***	8/1147	0.144 (0.052, 0.366)	***<0.001***
**I am afraid of the new COVID-19 variants:**						
Disagree or neutral	80/794	Reference		62/664	Reference	
Agree	25/892	0.257 (0.159, 0.402)	***<0.001***	21/751	0.644 (0.314, 1.299)	0.223

^a^ Univariate logistic regression analysis; ^b^ Multivariate logistic regression analysis; ^c^ Global *p*-values generated by likelihood ratio tests; OR, odds ratio; CI, confidence interval.
